# Using Long Short-Term Memory (LSTM) recurrent neural networks to classify unprocessed EEG for seizure prediction

**DOI:** 10.3389/fnins.2024.1472747

**Published:** 2024-11-15

**Authors:** Jordan D. Chambers, Mark J. Cook, Anthony N. Burkitt, David B. Grayden

**Affiliations:** ^1^Department of Biomedical Engineering, The University of Melbourne, Melbourne, VIC, Australia; ^2^Seer Medical, Melbourne, VIC, Australia; ^3^Departments of Medicine and Neurology, St Vincent's Hospital, The University of Melbourne, Melbourne, VIC, Australia; ^4^Graeme Clark Institute for Biomedical Engineering, The University of Melbourne, Melbourne, VIC, Australia

**Keywords:** epilepsy, EEG, seizure prediction, machine learning, long short-term memory, LSTM

## Abstract

**Objective:**

Seizure prediction could improve quality of life for patients through removing uncertainty and providing an opportunity for acute treatments. Most seizure prediction models use feature engineering to process the EEG recordings. Long-Short Term Memory (LSTM) neural networks are a recurrent neural network architecture that can display temporal dynamics and, therefore, potentially analyze EEG signals without performing feature engineering. In this study, we tested if LSTMs could classify unprocessed EEG recordings to make seizure predictions.

**Methods:**

Long-term intracranial EEG data was used from 10 patients. 10-s segments of EEG were input to LSTM models that were trained to classify the EEG signal. The final seizure prediction was generated from 5 outputs of the LSTM model over 50 s and combined with time information to account for seizure cycles.

**Results:**

The LSTM models could make predictions significantly better than a random predictor. When compared to other publications using the same dataset, our model performed better than several others and was comparable to the best models published to date. Furthermore, this framework could still produce predictions significantly better than chance when the experimental paradigm design was altered, without the need to reperform feature engineering.

**Significance:**

Removing the need to perform feature engineering is an advancement on previously published models. This framework can be applied to many different patients’ needs and a variety of acute interventions. Also, it opens the possibility of personalized seizure predictions that can be altered to meet daily needs.

## 1 Introduction

Seizure prediction could improve quality of life for patients through removing uncertainty and providing an opportunity for acute treatments. Consequently, seizure prediction has attracted a lot of interest, particularly since it was shown to be feasible [for examples, see (Iasemidis et al., 1990; Iasemidis et al., 2005; Lehnertz and Elger, 1998; Moser et al., 1999) or for more comprehensive reviews see (Iasemidis, 2011; Iasemidis and Sackellares, 1996; Kuhlmann et al., 2018b; Litt and Echauz, 2002)]. Significant progress in seizure prediction has been made due to improvements in computer technologies (Litt and Echauz, 2002), the accumulation of data (Kuhlmann et al., 2018b; Wong et al., 2023) and advances in machine learning algorithms (Maimaiti et al., 2022). The accumulation of data is not just due to more studies and more patients, but also the development of medical devices that allow long-term recordings (Andrzejak et al., 2023; Stirling et al., 2021). The creation of larger datasets means machine learning algorithms improve in performance.

The most common approach using machine learning, or similar optimization techniques, is to perform feature extraction on the EEG recordings. These features are then used as input into a model designed to make a prediction. This approach is efficient in terms of directing the model toward important information and in terms of computational costs for optimizing the model. However, the performance of the model is restricted by the features extracted and the feature extraction is a process that requires human intuition. Models developed this way are limited to the information captured by the features extracted. Any changes to the experiment paradigm may reduce the performance of the model if the new paradigm requires information not captured in the original extracted features. In seizure prediction, this is particularly important because there is no agreement in what the best prediction timeframe is (Arthurs et al., 2010; Schulze-Bonhage et al., 2010) and different acute treatments will require different time courses to be effective. Therefore, a good seizure prediction model should be able to alter the experimental paradigm (such as seizure prediction horizon (SPH) and intervention period) to be useful for a range of patient needs and a range of acute treatments.

Recurrent neural networks are a form of machine learning architecture that have a feedback loop. This allows information to persist and, therefore, display temporal dynamics. As such, recurrent neural networks are ideal to analyze sequences or time series data, like EEG recordings. Long Short-Term Memory (LSTM) neural networks (Hochreiter and Schmidhuber, 1997) are a special form of recurrent networks designed to deal with the vanishing gradient problem, which means they can learn long-term dependencies in the data.

Since LSTMs are well suited to interpret EEG, several studies have started to use LSTMs for seizure prediction [for example, (Ali et al., 2019; Daoud and Bayoumi, 2019; Ma et al., 2018; Pal Attia et al., 2023; Payne et al., 2023; Tsiouris et al., 2018; Varnosfaderani et al., 2021; Viana et al., 2023; Zhang et al., 2021)]. However, most these studies perform some sort of feature extraction on the EEG before and passing those features to the LSTM model. Again, this limits the prediction power of the LSTM model to the extracted features. For example, a common approach is to perform a Fourier transform on the EEG data and then pass the frequency-time data to the LSTM model (Ali et al., 2019; Ma et al., 2018; Pal Attia et al., 2023; Payne et al., 2023; Viana et al., 2023). This approach is efficient in terms of computational costs, but can lose important information, such as autocorrelation information [which has been shown to be a good predictor for seizures (Maturana et al., 2020)]. Using convolutional neural networks (or similar architecture like encoders or perceptron) to process the EEG recordings (Daoud and Bayoumi, 2019; Lopes et al., 2024; Zhou et al., 2018) has the potential to avoid losing such information, but it is unclear if performance is increased or decreased by having convolutional neural networks before or after the LSTM units.

In this current study, we used LSTMs to process raw EEG signals to make seizure predictions. Long-term intracranial EEG data was used (Cook et al., 2013). The LSTM models could make predictions significantly better than a random predictor and better than several studies using the same dataset. Furthermore, this framework could still produce good predictions when the experimental paradigm was altered, which is an improvement on previous models.

## 2 Methods

### 2.1 Data

Long-term intracranial EEG from the NeuroVista dataset was used (Cook et al., 2013). This consisted of 16 electrodes continuously recording at 400 Hz for 0.5–2.1 years (1.5 years on average) for 15 patients with refractory focal epilepsy. The clinical feasibility study and sharing of data was approved by the Human Research Ethics Committee, St. Vincent’s Hospital, Melbourne (approval LRR145/13).

Of the original 15 patients, 10 patients were used in this study. During model development, initial tests indicated approximately 30 seizures were required to train the LSTM models, which is similar to previous studies using the same dataset (Karoly et al., 2017; Kiral-Kornek et al., 2018; Kuhlmann et al., 2018a; Payne et al., 2023). Therefore, patients 4, 5, 12, and 14 were excluded because they had less than 15 seizures. Patient 7 was also excluded due to a combination of shorter recording time (less than 7 months) and only 35 lead seizures. The recording times and seizure counts are provided for all patients in the Supplementary information.

We only used lead seizures to train and test the algorithms, consistent with other studies (Kiral-Kornek et al., 2018; Kuhlmann et al., 2018a). A lead seizure was defined as a seizure that did not have any seizure in the 4 h prior. Similarly, 4 h of data after each seizure was excluded from the datasets to avoid post-ictal patterns.

The first 100 days of the recordings were excluded due to the inconsistency of the recordings (Ung et al., 2017). The remaining data was split into training and testing sets with an 80:20 split. The first 80% of seizures were allocated to the training set, so that there was no chance of time-correlated data being used in the test set (West et al., 2023). The half-way point between the last seizure in the training set and the first seizure in the test set was used to separate the two datasets.

The input into the model was 1 min of EEG recordings across 16 electrodes. The only pre-processing that was performed was a normalization of the amplitude and removal of NaNs (not a number), which were due to missing data (usually because of NeuroVista device telemetry drop-outs). No other preprocessing of data was performed such as artifact removal or control for changes in signal quality. The amplitude was normalized so the raw values would fall into a range that was well suited to the sigmoid activation function of the LSTM units. This normalization was done separately for each electrode and involved subtracting the mean value of the signal for that 1 min recording and dividing by the average standard deviation (where the average was calculated from the previous 30 days of recordings and was updated once per day). Model development indicated a better performance when using the average standard deviation compared to using the standard deviation of the 1 min segment of EEG because it included amplitude information in different brain states observed over many days. Missing data values were replaced with the mean value of the signal for that electrode and that 1 min recording. A 1 min recording was excluded from the dataset if there was more than 400 NaNs, or more than 1 s of data missing, which allowed for small data dropouts (for example, from telemetry interruptions) but excluded minutes where larger dropouts started and finished (for example, charging the device battery). All other 1 min recordings prior to a lead seizure were included in the dataset.

Balanced datasets were used to train the models, whereas unbalanced datasets were used to test the models. Two different datasets were used to train the model. The first dataset consisted of 10 s segments of raw EEG to train the LSTM units. Since inter-ictal data out-numbered pre-ictal data, we used up-sampling of the pre-ictal data to create a balanced dataset. EEG data was stored in files comprising 1-min recordings. The inter-ictal dataset was created by taking the first 10 s of every file that was labelled as inter-ictal. The pre-ictal dataset was created by taking multiple 10 s segments of every file to up sample this dataset. First, this was done with even spacing (for example, 0–10 s, 10–20 s, etc.), followed by overlapping with increased amounts of overlap (5–15 s, 15–25 s, etc., then 2.5–12.5 s, 12.5–22.5 s) and finally by using a random number generator to give the start time of the 10 s segment (at a resolution of 2.5 ms). In the cases where more than two labels were used (Paradigms 3 and 4), the same procedure was used to up-sample all labels to match the label with the highest number of files.

The second dataset used to train the model consisted of 1 min segments of EEG recordings. The parameters of the LSTM units were held constant while the classifier was trained. Up-sampling of this dataset was performed by creating random noise (± 5%) of the inputs into the classifier.

Unbalanced datasets were always used to test the models. To create the test datasets, for every file of 1-min of EEG recording, five 10 s samples were generated with no overlap (0–10 s, 10–20 s, etc.). A sixth sample was not created because it would require some level of overlap due to the final recorded sampling rate being just below 400 Hz (approximately 399.6 Hz).

Model development and hyperparameter exploration was performed with three patients (Patients 1, 6 and 13) using a subset of the training data. *None of the final test dataset data was accessed until the final models were run on the test data. Furthermore, no data from 7 out of 10 patients was accessed until the final training and test runs of these models.*

### 2.2 The model

**Figure 1:**
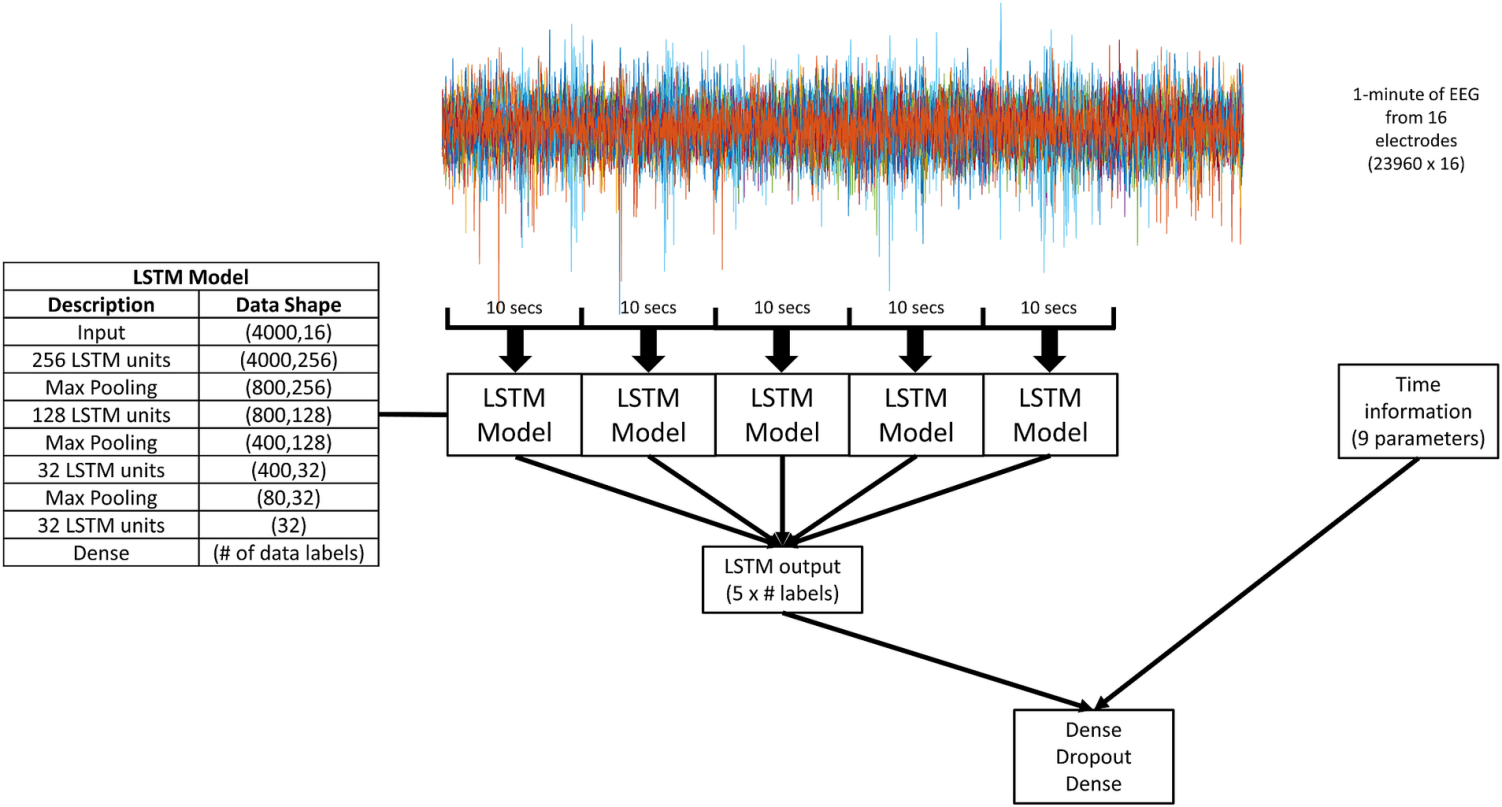
A schematic diagram of the overall model. 1 min segments of the EEG recordings at 400 Hz across 16 electrodes were used as the input. The 1 min recordings were broken up into five 10 s segments with no overlap. Each 10 s segment was played into the LSTM model (details given in the inserted table). The output of the LSTM model was two, four, or five values (depending on the number of data labels for that experimental paradigm, as indicated by “# labels” in the figure). The output of five LSTM models was combined with time information through a simple dense classifier. The dense classifier comprised of two dense layers separated by a dropout layer.

Figure 1 describes the model structure. 1 min segments of the EEG recordings at 400 Hz across 16 electrodes were used as the input into the model. The 1 min recordings were broken up into five 10 s segments with no overlap. Each 10 s segment was played into the LSTM model. Five outputs from the LSTM model were combined with time information through a simple classifier to generate a final forecast or prediction.

#### 2.2.1 LSTM model

The model development and a range of machine learning model structures tested are described in the Supplementary material. LSTM units were used to process 10 s segments of raw EEG recordings. LSTM units can process time series data through two state parameters and a series of three gates [for a full description see (Hochreiter and Schmidhuber, 1997)]. For each data point in the time series, the LSTM unit calculates the two state parameters using the three gates. The two state parameters are a cell state (*C_t_*) and a hidden state (*h_t_*). The cell state contains information that can be retained for many time steps. The hidden state produces the new output of the LSTM unit at each time step. The three gates are an input gate (*i_t_*), a forget gate (*f_t_*) and an output gate (*o_t_*). The input gate determines if the cell state should be updated with information from the current data point and the previous hidden state (Equation 1). The forget gate determines what to keep or forget from the current data point and previous hidden state (Equation 2). The output gate determines what information from the current data point and the previous hidden state is used to update the hidden state (Equation 3). Finally, the cell state and hidden state are updated. The cell state is updated by combining the output of the forget gate multiplied by the previous cell state with the output of the input gate multiplied by a potential new cell state (Equations 4 and 5). The hidden state is updated by combining the output gate with the cell state (Equation 6). For each gate, there are weights (W) and bias (U) parameters that are adjusted in training to learn the useful information required for seizure predictions. The equations to describing an LSTM unit are as follows:

(1) $$i_{t}=\sigma \left(x_{t}U^{i}+h_{t-1}W^{i}\right)$$

(2) $$f_{t}=\sigma \left(x_{t}U^{f}+h_{t-1}W^{f}\right)$$

(3) $$o_{t}=\sigma \left(x_{t}U^{o}+h_{t-1}W^{o}\right)$$

(4) $${\tilde{C}}_{t}=\tanh\left(x_{t}U^{g}+h_{t-1}W^{g}\right)$$

(5) $$C_{t}=\sigma \left(f_{t}*C_{t-1}+i_{t}*{\tilde{C}}_{t}\right)$$

(6) $$h_{t}=\tanh\left(C_{t}\right)*o_{t}$$

Where σ and *tanh* are the activation functions.

The model was made up of four LSTM layers, where each layer was separated by a max pooling layer, giving a total of three max pooling layers. The first three LSTM layers return the full sequence, so did not change the amount of data within the model. The max pooling layers were used to reduce the amount of data. Due to this reduction in data size, the number of LSTM units in each layer was reduced to speed up the training time. The final LSTM layer only returned a single value representing the final cell state. There was a final dense layer to reduce the number of variables within the model to be the same as the number of labels used in the data. For the LSTM layers, a sigmoid function was used for both the activation and recurrent activation. There was also a recurrent dropout (a dropout of the recurrent state during the linear transformation from one time step to the next) of 0.25. Adam (Kingma and Ba, 2014) was used as the optimizer with a learning rate of 10^−4^. Mean squared error was used as the loss function given that each label had its own output, which was set to zero or one.

#### 2.2.2 Combining LSTM model output with time information

Previously, it has been shown that seizures occur in cycles where the cycles can range from hours to months (Karoly et al., 2018; Karoly et al., 2017). It has also been shown that including time-of-day information with information from EEG improves seizure predictions (Kiral-Kornek et al., 2018). Therefore, we combined the output of the LSTM model with time information (Figure 1). For every 1 min of EEG recording, five 10 s samples with no overlap were selected and input to the LSTM model. The outputs from the five LSTM models were combined with time information. There were nine values for the time information:

- Two values for the hour of day (24 h cycle).
- Two values for the day of the month (31 day cycle).
- Two values for the month of the year (12 month cycle).
- Two values day of the week (7 day cycle).
- The log of the minutes since the last seizure (while not related to the time of day, this value was added to help the model identify cycles that did not easily fit into the four cycles relating to time, such as a 12 day cycle).

For each of the four cycles regarding time, two values were given. The first represented the actual value and the second represented the time period from the start of the cycle, to provide the cyclic information of time to the classifier. Both values were normalized to the range of 0–5. Both values were calculated with a resolution of 1 min, so that each 1 min file of EEG recording had a unique value for each parameter of the time information.

The output of the LSTM models and the time information was combined using a simple dense (or fully connected) classifier. This classifier comprised two dense layers separated by a dropout layer. The number of neurons in the first dense layer was 10 times the number of data labels. The dropout rate used was 0.25. The number of neurons in the second dense layers was equal to the number of data labels. A sigmoid activation function was used in both dense layers. Again, Adam was used as the optimizer with learning rate 10^−4^. Mean squared error was used as the loss function.

### 2.3 Experimental paradigms

Four different experimental paradigms were tested to see if the LSTM correctly classifies EEG signals with different labelling systems:

Paradigm 1: Pre-ictal was labelled as 1–16 min prior to seizure and inter-ictal was labelled as more than 16 min before a seizure. This matched the labelling used in the Deep CNN model (Kiral-Kornek et al., 2018). The Deep CNN model was chosen as a comparison because it was very similar to this model in terms of using machine learning algorithms with EEG data and time information.

Paradigm 2: Pre-ictal was labelled as 1–4 min prior to seizure and inter-ictal was labelled as more than 4 min before a seizure. This matched the labelling used in the critical slowing model (Maturana et al., 2020). The critical slowing model was chosen as a comparison because it has produced the best results to date.

Paradigm 3: EEG data was classified into four different labels relating to the time prior to seizure. Label 1 was 1–15 min before a seizure, label 2 was 15–75 min before a seizure, label 3 was 75 min-24 h before a seizure, and label 4 was more than 24 h before a seizure.

Paradigm 4: EEG data was classified into five different labels relating to the time prior to seizure. Label 1 was 1–5 min before a seizure, label 2 was 5–65 min before a seizure, label 3 was 65 min-8 h before a seizure, label 4 was 8–24 h before a seizure, and label 5 was more than 24 h before a seizure.

### 2.4 Statistics

To analyze the output from the experimental paradigms 1 and 2, we used the Receiver Operator Characteristic (ROC) curve. We calculated a linear combination of the two output values from the model (in paradigms 1 and 2) to create a single value as the receiver operator. The ROC curve characterizes the relationship between two parameters defined as the true positive rate (proportion of true positives to true positives plus false negative) and false positive rate (proportion of false positives to false positives plus true negatives). The Area under the Curve (AUC) measures the area underneath the entire ROC curve, with greater AUC representing better performance.

To compare AUCs, we calculated confidence intervals using the Hanley and McNeil method (Hanley and McNeil, 1983). A difference between AUCs was considered statistically significant when there was no overlap between the confidence intervals of both AUCs. When comparing an AUC to a random predictor, if the lower bound of the confidence interval was above 0.5 it was considered statistically significant.

## 3 Results

We created a framework using LSTMs to process raw EEG signals to make seizure predictions without the use of any feature engineering. The LSTM models make predictions well above a random predictor and better than several publications using the same dataset. Furthermore, this framework could still produce good predictions when the experimental paradigm design was altered.

### 3.1 LSTMs can classify unprocessed EEG recordings to predict seizures

To ensure the LSTMs could classify EEG signals, we created artificial classes of EEG data by inserting a marker (distinct signal of 25 milliseconds) into the EEG recordings of patients. We demonstrated that LSTMs could classify EEG these artificial classes (see Supplementary information). The model was then tested to classify pre-ictal versus inter-ictal for three patients, which it did well above chance. We improved the performance by incorporating time information (to assist the model in identifying seizure cycles) and LSTM predictions over five 10 s periods. We ran this final model using a test set of 10 patients, training on the first 80% of seizures and testing on the final 20% of seizures. *This meant the training data from seven patients had never been seen by our model and the test dataset had never been seen by any of our models.* All results presented below are from the final test dataset.

**Figure 2:**
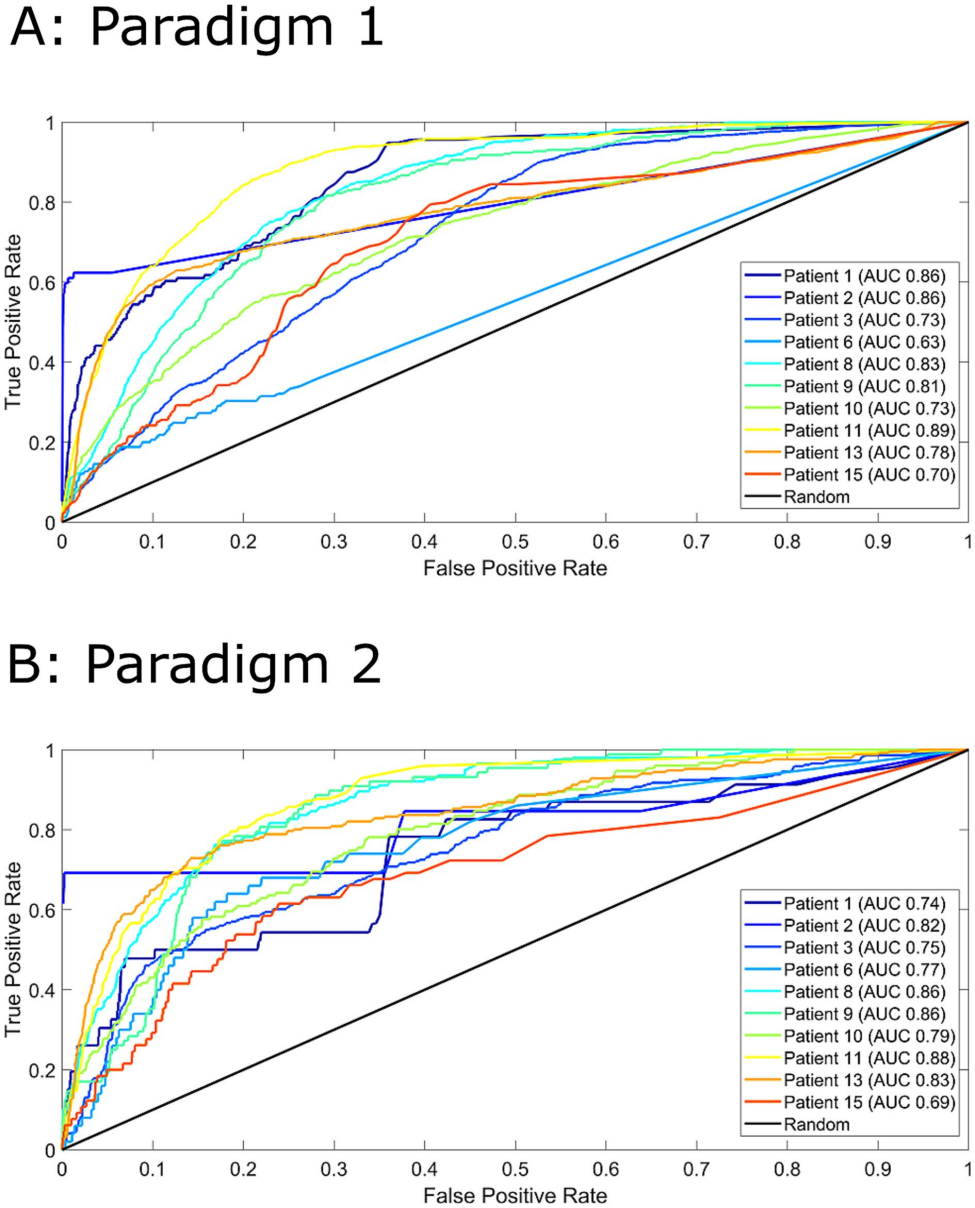
Receiver operator characteristics (ROC) curves for all 10 patients for paradigm 1 (Panel A) and paradigm 2 (Panel B). For paradigm 1 (Panel A), pre-ictal data was labelled as 1-16 minutes prior to seizure and inter-ictal as more than 16 minutes. For paradigm 2 (Panel B), pre-ictal data was labelled as 1-4 minutes prior to seizure and inter-ictal as more than 4 minutes. Area under the curve (AUC) values are indicated in the legend. A random predictor is indicated by the black line.

Figure 2 displays the ROC curves for 10 patients for paradigm 1 and 2. The model performed much better than a random predictor (Table 1). Comparing panels A and B in Figure 2 shows the effects of changing the data labels, where three patients show a statistically significant difference (Table 1). Patient 1 shows a significant decrease when using the 1–4 min prior to seizure, whereas Patients 6 and 13 show a significant increase when using the 1–4 min prior to seizure. It should also be noted that Patient 10 showed an increase that was almost significant.

**Table 1:** The AUC values and Hanley McNeil confidence intervals.

Patient	Paradigm 1 AUC	Paradigm 2 AUC	No overlap in the confidence interval (Paradigm 1 compared to Paradigm 2)
Patient 1	0.86 (±0.039)	0.74 (±0.083)	*
Patient 2	0.86 (±0.053)	0.82 (±0.100)	
Patient 3	0.73 (±0.019)	0.75 (±0.033)	
Patient 6	0.63 (±0.046)	0.77 (±0.077)	*
Patient 8	0.83 (±0.019)	0.86 (±0.032)	
Patient 9	0.81 (±0.029)	0.86 (±0.050)	
Patient 10	0.73 (±0.024)	0.79 (±0.043)	#
Patient 11	0.89 (±0.017)	0.88 (±0.031)	
Patient 13	0.78 (±0.019)	0.83 (±0.031)	*
Patient 15	0.70 (±0.039)	0.69 (±0.072)	

The AUC values and Hanley McNeil confidence intervals for all patients from experimental Paradigms 1 and 2. For Paradigm 1, pre-ictal was defined as 1–16 min before seizure. For Paradigm 2, pre-ictal was defined as 1–4 min before seizure. When comparing an AUC to a random predictor, if the lower bound of the confidence interval was above 0.5 it was considered statistically significant, which indicates at all results for Paradigm 1 and Paradigm 2 were significantly better than a random predictor. The fourth column indicates if there was no overlap in the confidence interval when comparing the results in Paradigm 1 with the results in Paradigm 2 (* indicates that there was no overlap, # indicates a minor overlap of 0.003).

### 3.2 Comparison to previously published models using the same dataset

Our model performed better than a random predictor, but there have been many studies using the same dataset that have also performed well [for example, (Chen et al., 2022; Cook et al., 2013; Karoly et al., 2018; Karoly et al., 2017; Kiral-Kornek et al., 2018; Maturana et al., 2020)]. To compare the performance of this model to previous models, we chose to two top-performing models, the Deep CNN model (Kiral-Kornek et al., 2018) and the critical slowing model (Maturana et al., 2020). Performance of these models was reported using the metrics sensitivity and time in high. Sensitivity represents the proportion of pre-ictal data correctly predicted. Time in high represents the proportion of all predictions that are labelled as pre-ictal. Therefore, desired performance is sensitivity as high as possible while keeping time in high as low as possible. Since sensitivity and time in high are a snapshot of the ROC curve at one location, to compare these results with our model, we looked up the closest matching sensitivity (which allowed a direct comparison of the time in highs) and the closest matching time in high (which allowed a direct comparison of the sensitivities). However, it should be noted that this is not a perfect comparison. Both previous studies are designed to process a continuous stream of data and flag a warning if a seizure is imminent. While this current algorithm classifies 1 min segments of EEG, it can readily be implemented to update this classification every 2.5 msec (or every time step), hereby producing the equivalent forecasts. Such an implementation makes very little difference in the results presented in this work because it just increases the number of samples without changing the data presented to the model. A major difference between this work and the previous models is the calculation of the sensitivity. Previous models have calculated sensitivity as a seizure level event. That is, if the model produces a warning once during the pre-ictal period it is considered to accurately predict that seizure. Whereas in this current work every sample in the pre-ictal period needs to be classified as pre-ictal to achieve the highest sensitivity, which is more difficult. Furthermore, calculating the sensitivity as a seizure level event can cause issues with calculating the time in high, unless the time in high is adjusted so that every seizure warning produces a time in high for the same duration has the length of the pre-ictal period. Without this adjustment, a discrepancy between the time scales for the sensitivity and time in high is introduced. This current work avoids such a discrepancy to accurately produce ROC curves.

Table 2 compares the results of the Deep CNN model with LSTM model for Paradigm 1. The columns under the LSTM heading represent values when the pre-ictal label was higher than the inter-ictal label or a threshold of 0.5 on the ROC curve. Matching the sensitivity or time in high to the published values in the Deep CNN paper allows a direct comparison, which shows the LSTM model performed better for Patients 1, 3, 8, 9, 15. Patient 2 did not provide a good match with either the sensitivity or time in high due to jumps in the values, but our model did provide a slightly lower sensitivity for a far lower time in high. For Patient 11, both models produced almost the same performance. The Deep CNN model performed better for Patients 10 and 13.

**Table 2:** Comparison between Deep CNN model (Kiral-Kornek et al., 2018) and Paradigm 1.

	Deep CNN		LSTM		LSTM with matched sensitivity		LSTM with matched time in high	
	Sensitivity	Time in High	Sensitivity	Time in High	Sensitivity	Time in High	Sensitivity	Time in High
**Patient 1**	**0.65**	**0.21**	**0.39**	**0.02**	**0.65**	**0.18**	**0.69**	**0.21**
Patient 2	0.74	0.11	0.57	0.002	0.62	0.01	0.62	0.06
**Patient 3**	**0.71**	**0.53**	**0.69**	**0.39**	**0.71**	**0.41**	**0.89**	**0.53**
Patient 6			0.18	0.06				
**Patient 8**	**0.77**	**0.32**	**0.78**	**0.21**	**0.77**	**0.25**	**0.84**	**0.32**
**Patient 9**	**0.83**	**0.43**	**0.81**	**0.30**	**0.83**	**0.32**	**0.90**	**0.43**
Patient 10	0.68	0.32	0.50	0.18	0.68	0.36	0.64	0.32
Patient 11	0.78	0.18	0.62	0.09	0.78	0.17	0.79	0.18
Patient 13	0.70	0.21	0.63	0.14	0.70	0.25	0.68	0.21
**Patient 15**	**0.59**	**0.37**	**0.04**	**0.008**	**0.59**	**0.28**	**0.72**	**0.37**

Comparison of the results of the Deep CNN model (Kiral-Kornek et al., 2018) with the LSTM model for Paradigm 1. The columns under the LSTM heading represent the sensitivity and time in high for when the pre-ictal label was higher than the inter-ictal label or a threshold of 0.5 on the ROC curve. Matching the sensitivity or time in high to the published values in the Deep CNN paper allows a direct comparison between the results. When the current LSTM model performed better than Deep CNN model values are highlighted in bold.

Table 3 compares the results of the critical slowing model with the LSTM model for Paradigm 2. Again, matching the sensitivity or time in high shows the critical slowing model performed better for Patients 1, 2, 6, 9, 10, 11 and 15. The LSTM model performed better for Patients 8 and 13.

**Table 3:** Comparison between critical slowing model (Maturana et al., 2020) and Paradigm 2.

	Critical slowing		LSTM		LSTM with matched sensitivity		LSTM with matched time in high	
	Sensitivity	Time in High	Sensitivity	Time in High	Sensitivity	Time in High	Sensitivity	Time in High
Patient 1	0.83	0.08	0.22	0.02	0.83	0.42	0.48	0.08
Patient 2	0.87	0.0002	0.69	0.004	0.85	0.38	0.61	0.001
Patient 3			0.49	0.12				
Patient 6	0.66	0.03	0.28	0.06	0.66	0.21	0.08	0.03
**Patient 8**	**0.64**	**0.23**	**0.78**	**0.20**	**0.64**	**0.12**	**0.81**	**0.23**
Patient 9	0.85	0.16	0.84	0.26	0.85	0.26	0.72	0.16
Patient 10	0.78	0.24	0.58	0.17	0.78	0.34	0.64	0.24
Patient 11	0.86	0.16	0.38	0.04	0.86	0.26	0.72	0.16
**Patient 13**	**0.64**	**0.14**	**0.75**	**0.17**	**0.64**	**0.10**	**0.72**	**0.14**
Patient 15	0.87	0.0007	0.08	0.01	0.83	0.73	0.03	0.0007

Compares the results of the critical slowing model (Maturana et al., 2020) with the LSTM model for paradigm 2. The columns under the LSTM heading represent the sensitivity and time in high for when the pre-ictal label was higher than the inter-ictal label or a threshold of 0.5 on the ROC curve. Matching the sensitivity or time in high to the published values in the critical slowing paper allows a direct comparison between the results. When the current LSTM model performed better than critical slowing model, values are highlighted in bold.

### 3.3 Changing the experimental paradigm to a multi-class system using four and five data labels

Since our LSTM model can process raw EEG and classify pre-ictal versus inter-ictal, we tested to see if the model can classify raw EEG into more than two categories. This is like the original clinical feasibility study, where the patient advisory system indicated the seizure risk as low, moderate, or high. In this study, we labelled the data according to the time prior to seizure using either a four-label system or a five-label system, as described in paradigms 3 and 4.

For both paradigms 3 and 4, the LSTM model was able to process the raw EEG and classify it into one of the labels with much better accuracy than a random predictor. The confusion matrixes of 10 patients for paradigms 3 and 4 are provided in the Supplementary information. All patients showed a total accuracy above a random predictor for paradigm 3 (0.33–0.83, range of total accuracy across all patients) and paradigm 4 (0.27–0.78, range of total accuracy across all patients). The large range observed in the total accuracy was due to changes in performance of labels with a large proportion of the data (for example, comparing labels 8–24 h prior to seizure with more than 24 h). Furthermore, the proportion of samples selected for each label showed proportions like the actual proportions of the test dataset. Given the model was trained on a balanced dataset, these results indicate the model was working well and far better than a random predictor. Taking the total accuracy and the proportion of time each label was selected together, the model performed many times better than a random predictor.

The results could also be compared to a random predictor with the same time selected for each label. Taking Patient 1 in paradigm 3 as an example, a random predictor that selects 1–15 min prior to seizure 0.021 of the time, would have a sensitivity of 0.021 (when normalized along the actual row), whereas our model has a higher sensitivity of 0.364. Performing the same comparison for all labels over all patients for paradigm 3, this model performed better than a random predictor with the same time selections for 95% of labels and more than 0.1 better for 50% of labels. Similarly, for paradigm 4, this model performed better for 88% of labels and more than 0.1 higher for 56% of labels.

Therefore, the performance of the LSTM model was better than a random predictor for paradigms 3 and 4, but not as good as the performance for paradigms 1 and 2. Whilst this is expected due to the larger number of labels, providing more labels means the model is producing more information, which could be used by patients to meet their personal needs. We tested this idea by having the classifier (Figure 1) train and predict on a new set of data labels (dLSTM). In this case, the classifier was trained on data labelled as pre-ictal when 40–80 min prior to seizure and inter-ictal for more than 80 min prior to seizure. Table 4 shows the AUCs for these two models and for the CNN-LSTM model (Payne et al., 2023) with the same pre-ictal and inter-ictal definition. These results again show the seizure predictions were well above a random predictor (which would have an AUC of 0.5). For the dLSTMs trained on four labels, three patients had statistically significant increases in the AUC compared to the CNN-LSTM model and one patient had a statistically significant decrease. For the dLSTMs trained on five labels, two patients showed a statistically significant improvement in AUC compared to the CNN-LSTM model.

**Table 4:** Comparison between two dLSTM models and CNN-LSTM model (Payne et al., 2023).

	dLSTM with 4 labels	dLSTM with 5 labels	CNN-LSTM
Patient 1	0.83 (±0.028)^*^	0.79 (±0.030)	0.75 (±0.032)
Patient 2	0.61 (±0.047)	0.80 (±0.041)	
Patient 3	0.70 (±0.015)	0.70 (±0.015)	
Patient 6	0.58 (±0.030)	0.62 (±0.030)	0.64 (±0.030)
Patient 8	0.74 (±0.015)	0.73 (±0.015)	0.76 (±0.015)
Patient 9	0.83 (±0.019)	0.83 (±0.019)	0.80 (±0.020)
Patient 10	0.79 (±0.014)^*^	0.78 (±0.014)^*^	0.68 (±0.015)
Patient 11	0.81 (±0.015)	0.81 (±0.015)	0.82 (±0.014)
Patient 13	0.66 (±0.014)^*^	0.71 (±0.014)^*^	0.58 (±0.014)
Patient 15	0.65 (±0.026)^^^	0.67 (±0.026)	0.70 (±0.026)

AUCs for two LSTM models and CNN-LSTM model (Payne et al., 2023). The dLSTM models were variations on the LSTM with 4 or 5 labels, where the classifier was converted to make two predictions, pre-ictal or inter-ictal. Data was labelled as pre-ictal when 40–80 min prior to seizure and inter-ictal for more than 80 min prior to seizure, which matches these results from the CNN-LSTM model. Statistically significant difference was determined by no overlap in the Hanley and McNeil confidence intervals (which are the values displayed in the brackets).

* Indicates a statistically significant increase in performance when compared to the CNN-LSTM model.

^ Indicates a statistically significant decrease in performance when compared to CNN-LSTM model.

## 4 Discussion

The ability to predict when seizures will occur in patients with epilepsy could be life changing for these patients as it could remove uncertainty and potentially allow acute treatments to prevent seizures. As a result, there have been many studies looking at seizure prediction. Most of these algorithms involve feature extraction from the EEG recordings, which is an efficient process but comes at the cost of limiting the usefulness of the model, particularly when there are changes to prediction requirements, such as an increase in the SPH to allow for different interventions. To overcome these limitations, we developed a framework where machine learning algorithms process the raw EEG data to make seizure predictions.

We have demonstrated that LSTMs can process raw EEG recordings and classify the EEG recordings to make seizure predictions or forecasts. Often a forecast is defined to be a probability of a seizure occurring sometime in the future, whereas a prediction is defined to be pre-ictal or not. The output of our model is a probability of each data label, which is then converted to a single prediction so that it can be evaluated against the true data label. These seizure predictions were far better than a random predictor for four different experimental paradigms, which indicates that this framework could be readily used for patients requiring different types of seizure predictions and/or patients using different acute intervention. This is an improvement on previous work because usually prediction algorithms are not tested against multiple SPH and sometimes fail to perform as well when using different data and/or different experimental paradigms. It is likely that this improvement arises from the LSTMs learning to extract features itself. A preliminary investigation into what information the LSTMs are extracting from the EEG recordings indicates that sometimes the upstroke of a single oscillation is important, sometimes the peak of a single oscillation is important and sometimes information spread over many oscillations is important. However, further investigations are required to provide a detailed analysis of what information LSTMs are using to make seizure predictions.

Since all model development and validation was performed on a subset of training data from three patients, this framework had never seen any data from 70% of patients until the final training and testing evaluation. Therefore, it is expected this model would produce similar performance on any new data that provides the same information. Furthermore, it is reasonable to expect this framework to work on any intracranial EEG recordings and any data labelling. While LSTMs do require substantial computing resources to train, in a clinical setting the training time for an individual patient could be reduced to a day or two by distributing individual samples within a training batch on a high-performance computing facility. A limitation of this current work is using 80% of the EEG data to train the LSTM models. This limitation could be overcome by using an adaptive approach where model parameters are updated after each seizure (Iasemidis et al., 2005; Karoly et al., 2017) or by using patient independent models (Pal Attia et al., 2023).

### 4.1 Comparison to previously published model of seizure prediction

To compare our current model with previously published models, we only considered models that had used the same NeuroVista data (Cook et al., 2013). We did this because the NeuroVista dataset is the only long-term intracranial dataset currently available. Short-term datasets can have issues such as clinicians provoking seizures or low seizure numbers requiring pooling of data across patients (Freestone et al., 2017), time correlated data (West et al., 2023), and not being long enough to capture seizure cycles (Karoly et al., 2018; Karoly et al., 2017). As new devices become clinically available, such as NeuroPace (Razavi et al., 2020), analyzing long-term intracranial EEG recordings is going to become more important.

We did a direct comparison with two previously published models, the Deep CNN model (Kiral-Kornek et al., 2018) and the critical slowing model (Maturana et al., 2020). Whilst these comparisons are not perfect in terms of calculating the model performances as sensitivity and time in high, they do provide an indication of the relative performance. The Deep CNN model was used because of its similarity in only using EEG data and time information. Our model outperformed the Deep CNN model for six patients, one patient was essentially identical, and for two patients the Deep CNN model performed better. The Deep CNN model converted EEG recordings into the frequency-time domain, which is a very common approach in analyzing EEG. Our result of better performance using raw EEG compared to converting EEG to the frequency domain is the opposite of previous published findings (Zhou et al., 2018), which indicates the importance of using LSTMs to process the raw EEG as opposed to convolutional neural networks. These results suggest using a Fourier transform loses information that can improve seizure predictions (for example, changes in the autocorrelation). Therefore, allowing the machine learning algorithms access to the raw data provides a better framework for seizure prediction compared to extracting features that may only be relevant for one length of SPH.

The critical slowing model outperformed our model for seven patients, whereas our model performed better for two patients. Performing better for two patients is an achievement because, when the critical slowing model was published, it outperformed all previous models for the 14 patients it used. This would make our current LSTM model the best model published to date for two patients. It should be noted, the calculations of model sensitivity in this current work requires all pre-ictal samples to be classified as pre-ictal to achieve the highest sensitivity, where the critical slowing model only required one warning per pre-ictal period to achieve the highest sensitivity. Similarly, due to the data labelling in this current work, the seizure occurrence period (SOP) was only 1-min, whereas most previous studies have a much larger SOP.

Furthermore, the critical slowing model used several carefully selected features of the EEG data in combination with the seizure cycles. While this produces the best performance, it does raise questions about how the model will perform under different conditions. For example, the autocorrelation, a feature in the model, only changes less than 2–3 min before a seizure. If a patient requires more than 2–3 min of warning before a seizure, it is unknown how this model will perform because it will be relying on other features. We have shown our current LSTM model can easily predict seizures under different conditions (such as changing the SPH and changing the number of data labels), which is an improvement on previously published models.

The only other model that has demonstrated flexibility in prediction times is the CNN-LSTM model (Payne et al., 2023). In addition to flexibility, this model was also able to predict seizures with a seizure prediction horizon (the time between the warning and the seizure onset) of 40 min, the largest of any models using the NeuroVista dataset. We replicated this in our model by using the output from the four-label or five-label LSTMs and changing the data labels for the classifier. Our model demonstrated a statistically significant improvement for two patients while there was no statistical difference between the remaining patients. This demonstrates the good performance of our framework and indicates this framework can be easily and quickly adapted to meet unique requirements for individual patients. Training the LSTM models takes considerable time, but training the classifier can be done in 10 min on a stand-alone computer. This raises the possibility that individual patients could quickly and easily adapt an advisory system to their needs for that day.

Comparing our results to the Deep CNN model and the critical slowing model, it appears the performance of our LSTM model is comparable to the best models published to date, particularly when considering the differences in calculating the sensitivity. However, we have not made a direct comparison to several other models using the NeuroVista dataset [for example, (Chen et al., 2022; Cook et al., 2013; Karoly et al., 2017; Kuhlmann et al., 2018a)]. Our first and second experimental paradigms were deliberately chosen to be the same as those used for the Deep CNN model and the critical slowing model. When comparing the results of our model for these two experimental paradigms, three out of 10 patients showed statistically significant differences and a fourth patient the confidence intervals only just overlapped, which implies that a less stringent statistical test would have found a significant difference. Therefore, using the exact same prediction framework with the same data (except for a small change in the definition of the pre-ictal times) has produced significantly different results for at least 30% of patients. Given this result, it does not seem reasonable to compare the performance of different models when the data is labelled differently and handled differently (for example, selection criteria, pre-processing methods, normalization techniques, etc.). Ideally, there would be a standardized process for labelling and handling data for seizure predictions to allow direct comparisons between different studies. Indeed, this has been previously suggested (Kuhlmann et al., 2018a; Wiener et al., 2016). However, it would be very difficult to define such parameters because individual patients have different requirements for seizure predictions (Arthurs et al., 2010; Schulze-Bonhage et al., 2010) as will different intervention techniques. Therefore, a framework that allows for changes in the seizure prediction requirements, such as the one presented here, has the potential to be beneficial to a wider range of patients.

## 5 Conclusion

LSTMs can classify unprocessed EEG recordings to make seizure predictions better than chance and comparable performance to the best models. This framework produces good performance when the experimental design was altered, without the need to reperform feature engineering or alter the model structure. This is a significant advancement on previous works. Our framework was developed using minimal data and transferred well to unseen data and unseen patients. Therefore, it is expected this framework will perform well on new datasets and could be applied to other classification problems.

## Data Availability

Publicly available datasets were analyzed in this study. This data can be found here: all source code and trained models are freely available for download at https://github.com/JordanChambers/LSTM-seizure-prediction-raw-EEG. Patient data can be accessed at epilepsyecosystem.org. Patient data not available at epilepsyecosystem.org may be made available on request at epilepsyecosystem.org or by contacting the authors of the original clinical feasibility study (Cook et al., 2013).
