# Supplementary material for: Using Long Short-Term Memory (LSTM) recurrent neural networks to classify unprocessed EEG for seizure prediction
Source: Front Neurosci. 2024 Nov 15;18:1472747. doi: 10.3389/fnins.2024.1472747 (PMC11604808; doi:10.3389/fnins.2024.1472747)
Supplement: Supplementary file 1 [file Data_Sheet_1.docx]

Supplementary Material

# Seizure numbers and recording times

| **Patient** | **# days recorded** | **Total seizures** | **Seizures after 100 days** | **Lead seizures** |
| --- | --- | --- | --- | --- |
| Patient 1 | 767 | 152 | 120 | 79 |
| Patient 2 | 730 | 33 | 28 | 28 |
| Patient 3 | 557 | 389 | 325 | 232 |
| Patient 4 | 232 | 22 | 14 | 14 |
| Patient 5 | 272 | 8 | 4 | 4 |
| Patient 6 | 441 | 99 | 53 | 49 |
| Patient 7 | 184 | 336 | 116 | 35 |
| Patient 8 | 558 | 472 | 309 | 262 |
| Patient 9 | 394 | 212 | 165 | 161 |
| Patient 10 | 373 | 565 | 475 | 219 |
| Patient 11 | 721 | 446 | 343 | 289 |
| Patient 12 | 728 | 87 | 6 | 6 |
| Patient 13 | 746 | 501 | 470 | 345 |
| Patient 14 | 627 | 15 | 12 | 12 |
| Patient 15 | 465 | 77 | 61 | 61 |

Table S1: Shows the length of the recording time and seizure numbers for each patient. The first 100 days of recording are excluded due to variability in the recording. Lead seizures are defined as a seizure that did not have any seizure in the four hours prior.

# Model development

To test if LSTMs could perform pattern recognition on unprocessed EEG signals, we inserted markers into random EEG recordings from patients to create labels for EEG classification. A marker was a distinct signal of 25 milliseconds. For example, a marker that we tested was a straight line with value 1 or -1. We tested several markers including a straight line at the constant value, an inclining or declining straight line, a normal or upside-down V (Figure S1), and multiple oscillations with a fixed starting point and fixed ending point. We found that multiple model structures of LSTMs, similar recurrent networks, and traditional convolutional neural networks could identify the marker and correctly classify the data. The results for 25 different model structures are displayed in Table S1. To produce Table S1, 625 milliseconds of EEG recording were randomly chosen from 1 patient and a normal or upside-down V (Figure S1) was inserted at a random location.

| Model Description | V-shape  Test accuracy (%) |
| --- | --- |
| 2 LSTM | 51.0 |
| 16 LSTM; 2 dense | 55.3 |
| 64 LSTM; 2 dense | 94.2 |
| 128 LSTM; 2 dense | 96.9 |
| 16 LSTM; 16 LSTM; 2 dense | 57.0 |
| 16 LSTM; 16 LSTM; 16 LSTM; 2 dense | 61.6 |
| 16 LSTM; 16 LSTM; 16 LSTM; 16 LSTM; 2 dense | 77.9 |
| 16 LSTM; 16 LSTM; 16 LSTM; 16 LSTM; 16 LSTM; 2 dense | 61.7 |
| 16 LSTM; 16 biLSTM; 2 dense | 55.1 |
| 16 LSTM; 16 biLSTM; 16 biLSTM; 2 dense | 83.3 |
| 16 LSTM; 16 biLSTM; 16 biLSTM; 16 biLSTM; 2 dense | 94.1 |
| 16 GRU; 16 GRU; dense | 99.5 |
| 16 GRU; 16 GRU; 16 GRU; dense | 99.7 |
| 16 GRU; 16 GRU; 16 GRU; 16 GRU; dense | 99.5 |
| 16 LSTM; AvePool; 16 LSTM; AvePool; 16 LSTM; AvePool; 16 LSTM; 2 dense | 69.6 |
| 16 LSTM; MaxPool; 16 LSTM; MaxPool;16 LSTM; MaxPool; 16 LSTM; 2 dense | 90.3 |
| 16 LSTM; Conv1d; 16 LSTM; Conv1d; 16 LSTM; Conv1d; 16 LSTM; 2 dense | 54.4 |
| 32 LSTM; AvePool; 16 LSTM; AvePool; 8 LSTM; AvePool; 8 LSTM; 2 dense | 96.5 |
| 32 LSTM; MaxPool; 16 LSTM; MaxPool; 8 LSTM; MaxPool; 8 LSTM; 2 dense | 94.0 |
| 32 LSTM; Conv1d; 16 LSTM; Conv1d; 8 LSTM; Conv1d; 8 LSTM; 2 dense | 52.0 |
| 250 dense; 2 dense | 55.0 |
| 250 dense; 50 dense; 2 dense | 54.8 |
| 250 dense; 125 dense; 60 dense; 2 dense | 54.5 |
| 200 dense; 150 dense; 100 dense; 50 dense; 2 dense | 55.4 |
| conv1d; conv1d; conv1d; conv1d; 2 dense | 50.3 |
| conv1d; 125 dense; conv1d; 60 dense; conv1d; 30 dense; conv1d; 2 dense | 54.4 |

Table S2: Shows the test accuracy for EGG data labelled by inserting a biomarker for a variety of model structures. In the model description, sequential layers are separated by a colon, the numbers indicate number of units in that layer and the name indicates the type of layer in Tensorflow. The results are for 625 milliseconds of EEG recording were randomly chosen from 1 patient and a normal or upside-down V (Figure S1) was inserted at a random location. EEG data used for the final test accuracy was generated from different EEG data used in training.

Extending the results in Table S2, we found the LSTM models could still correctly identify the artificial classes when the EEG data was extended to 10 s and 16 electrodes. To make it easier for the model, four markers of 25 ms were inserted at random locations (similar results observed when the marker was increased in size to 100 ms). The model was able to correctly identify the artificial labels when the markers were inserted into all electrodes or even just one randomly selected electrode.

## Figure S1


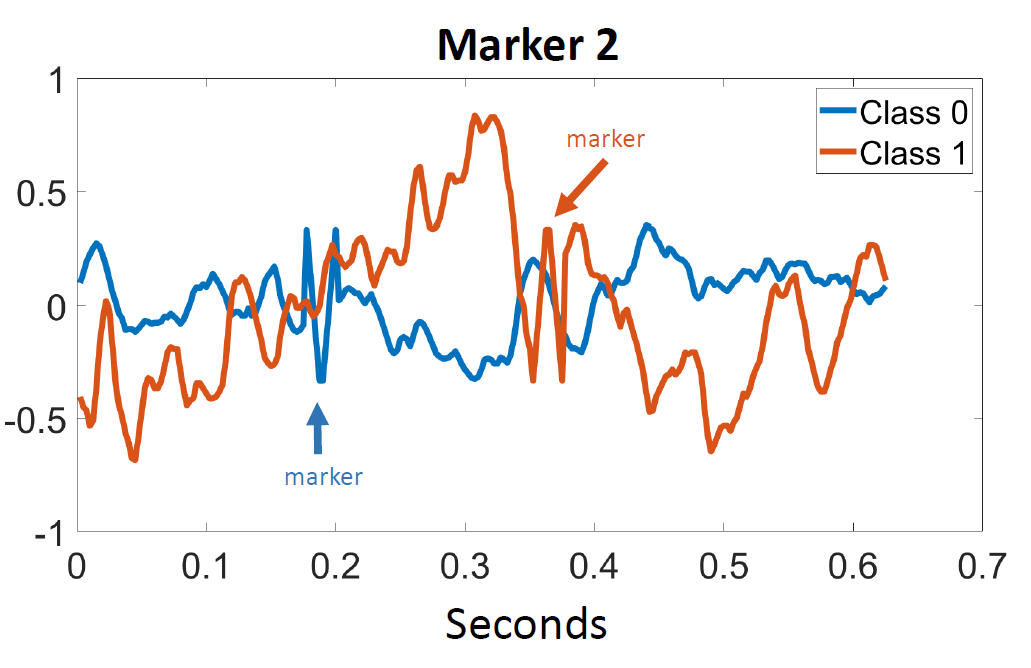


Figure S1: Example EEG recording with a marker inserted to generate artificial classes of EEG recordings. In this example, a V or up-side-down V was inserted into the EEG recordings.

## Multi-class labelling: four labels

Below are the confusion matrixes for a multi-class system. The labels were generated according to the time prior to a seizure. The four labels were 0: 1-15 min before a seizure; 1: 15-75 min before a seizure; 2: 75 min to 24 hr before a seizure; 3: more than 24 hr before a seizure.

| **Patient 1** | | **Predicted** | | | |
| --- | --- | --- | --- | --- | --- |
|  |  | **0** | **1** | **2** | **3** |
| **Actual** | **0 (0.001)** | 0.364 | 0.341 | 0.085 | 0.209 |
|  | **1 (0.005)** | 0.090 | 0.319 | 0.218 | 0.373 |
|  | **2 (0.085)** | 0.024 | 0.081 | 0.407 | 0.487 |
|  | **3 (0.909)** | 0.020 | 0.057 | 0.210 | 0.714 |
| **Time selected in predictions** | | 0.021 | 0.060 | 0.227 | 0.692 |
| **Proportion of total labels correct** | | 0.685 | | | |

Table S3: The confusion matrix for Patient 1 when using a four label system. Values in the matrix are normalized along the actual rows. Values in the brackets in the actual labels indicate the proportion of actual labels in the test data.

| **Patient 2** | | **Predicted** | | | |
| --- | --- | --- | --- | --- | --- |
|  |  | **0** | **1** | **2** | **3** |
| **Actual** | **0 (0.001)** | 0.507 | 0.068 | 0.110 | 0.315 |
|  | **1 (0.003)** | 0.093 | 0.110 | 0.097 | 0.700 |
|  | **2 (0.062)** | 0.005 | 0.023 | 0.127 | 0.845 |
|  | **3 (0.934)** | 0.001 | 0.012 | 0.102 | 0.885 |
| **Time selected in predictions** | | 0.002 | 0.013 | 0.104 | 0.881 |
| **Proportion of total labels correct** | | 0.835 | | | |

Table S4: The confusion matrix for Patient 2 when using a four label system. Values in the matrix are normalized along the actual rows. Values in the brackets in the actual labels indicate the proportion of actual labels in the test data.

| **Patient 3** | | **Predicted** | | | |
| --- | --- | --- | --- | --- | --- |
|  |  | **0** | **1** | **2** | **3** |
| **Actual** | **0 (0.029)** | 0.180 | 0.419 | 0.278 | 0.123 |
|  | **1 (0.081)** | 0.169 | 0.382 | 0.336 | 0.114 |
|  | **2 (0.653)** | 0.068 | 0.213 | 0.450 | 0.269 |
|  | **3 (0.236)** | 0.096 | 0.148 | 0.480 | 0.276 |
| **Time selected in predictions** | | 0.086 | 0.217 | 0.443 | 0.254 |
| **Proportion of total labels correct** | | 0.395 | | | |

Table S5: The confusion matrix for Patient 3 when using a four label system. Values in the matrix are normalized along the actual rows. Values in the brackets in the actual labels indicate the proportion of actual labels in the test data.

| **Patient 6** | | **Predicted** | | | |
| --- | --- | --- | --- | --- | --- |
|  |  | **0** | **1** | **2** | **3** |
| **Actual** | **0 (0.002)** | 0.026 | 0.192 | 0.314 | 0.468 |
|  | **1 (0.007)** | 0.032 | 0.257 | 0.298 | 0.413 |
|  | **2 (0.129)** | 0.022 | 0.150 | 0.411 | 0.417 |
|  | **3 (0.862)** | 0.025 | 0.069 | 0.241 | 0.665 |
| **Time selected in predictions** | | 0.025 | 0.081 | 0.263 | 0.631 |
| **Proportion of total labels correct** | | 0.628 | | | |

Table S6: The confusion matrix for Patient 6 when using a four label system. Values in the matrix are normalized along the actual rows. Values in the brackets in the actual labels indicate the proportion of actual labels in the test data.

| **Patient 8** | | **Predicted** | | | |
| --- | --- | --- | --- | --- | --- |
|  |  | **0** | **1** | **2** | **3** |
| **Actual** | **0 (0.010)** | 0.566 | 0.148 | 0.102 | 0.184 |
|  | **1 (0.034)** | 0.438 | 0.329 | 0.096 | 0.137 |
|  | **2 (0.446)** | 0.178 | 0.192 | 0.508 | 0.122 |
|  | **3 (0.510)** | 0.161 | 0.177 | 0.496 | 0.165 |
| **Time selected in predictions** | | 0.182 | 0.189 | 0.484 | 0.145 |
| **Proportion of total labels correct** | | 0.33 | | | |

Table S7: The confusion matrix for Patient 8 when using a four label system. Values in the matrix are normalized along the actual rows. Values in the brackets in the actual labels indicate the proportion of actual labels in the test data.

| **Patient 9** | | **Predicted** | | | |
| --- | --- | --- | --- | --- | --- |
|  |  | **0** | **1** | **2** | **3** |
| **Actual** | **0 (0.016)** | 0.385 | 0.431 | 0.184 | 0.000 |
|  | **1 (0.055)** | 0.216 | 0.540 | 0.237 | 0.007 |
|  | **2 (0.473)** | 0.096 | 0.163 | 0.738 | 0.004 |
|  | **3 (0.457)** | 0.121 | 0.185 | 0.658 | 0.035 |
| **Time selected in predictions** | | 0.119 | 0.198 | 0.665 | 0.018 |
| **Proportion of total labels correct** | | 0.401 | | | |

Table S8: The confusion matrix for Patient 9 when using a four label system. Values in the matrix are normalized along the actual rows. Values in the brackets in the actual labels indicate the proportion of actual labels in the test data.

| **Patient 10** | | **Predicted** | | | |
| --- | --- | --- | --- | --- | --- |
|  |  | **0** | **1** | **2** | **3** |
| **Actual** | **0 (0.010)** | 0.302 | 0.224 | 0.321 | 0.154 |
|  | **1 (0.044)** | 0.177 | 0.430 | 0.293 | 0.100 |
|  | **2 (0.572)** | 0.086 | 0.118 | 0.562 | 0.234 |
|  | **3 (0.373)** | 0.048 | 0.088 | 0.324 | 0.539 |
| **Time selected in predictions** | | 0.078 | 0.122 | 0.459 | 0.341 |
| **Proportion of total labels correct** | | 0.545 | | | |

Table S9: The confusion matrix for Patient 10 when using a four label system. Values in the matrix are normalized along the actual rows. Values in the brackets in the actual labels indicate the proportion of actual labels in the test data.

| **Patient 11** | | **Predicted** | | | |
| --- | --- | --- | --- | --- | --- |
|  |  | **0** | **1** | **2** | **3** |
| **Actual** | **0 (0.009)** | 0.468 | 0.322 | 0.147 | 0.063 |
|  | **1 (0.030)** | 0.115 | 0.442 | 0.293 | 0.150 |
|  | **2 (0.434)** | 0.044 | 0.120 | 0.639 | 0.198 |
|  | **3 (0.527)** | 0.049 | 0.163 | 0.517 | 0.271 |
| **Time selected in predictions** | | 0.052 | 0.154 | 0.560 | 0.234 |
| **Proportion of total labels correct** | | 0.438 | | | |

Table S10: The confusion matrix for Patient 11 when using a four label system. Values in the matrix are normalized along the actual rows. Values in the brackets in the actual labels indicate the proportion of actual labels in the test data.

| **Patient 13** | | **Predicted** | | | |
| --- | --- | --- | --- | --- | --- |
|  |  | **0** | **1** | **2** | **3** |
| **Actual** | **0 (0.006)** | 0.557 | 0.114 | 0.098 | 0.231 |
|  | **1 (0.020)** | 0.167 | 0.220 | 0.260 | 0.353 |
|  | **2 (0.364)** | 0.107 | 0.070 | 0.288 | 0.534 |
|  | **3 (0.610)** | 0.143 | 0.034 | 0.295 | 0.528 |
| **Time selected in predictions** | | 0.133 | 0.052 | 0.290 | 0.525 |
| **Proportion of total labels correct** | | 0.435 | | | |

Table S11: The confusion matrix for Patient 13 when using a four label system. Values in the matrix are normalized along the actual rows. Values in the brackets in the actual labels indicate the proportion of actual labels in the test data.

| **Patient 15** | | **Predicted** | | | |
| --- | --- | --- | --- | --- | --- |
|  |  | **0** | **1** | **2** | **3** |
| **Actual** | **0 (0.002)** | 0.019 | 0.043 | 0.159 | 0.778 |
|  | **1 (0.009)** | 0.003 | 0.028 | 0.122 | 0.847 |
|  | **2 (0.174)** | 0.014 | 0.044 | 0.124 | 0.818 |
|  | **3 (0.815)** | 0.009 | 0.020 | 0.144 | 0.827 |
| **Time selected in predictions** | | 0.010 | 0.025 | 0.141 | 0.825 |
| **Proportion of total labels correct** | | 0.696 | | | |

Table S12: The confusion matrix for Patient 15 when using a four label system. Values in the matrix are normalized along the actual rows. Values in the brackets in the actual labels indicate the proportion of actual labels in the test data.

## Multi-class labelling: five labels

Below are the confusion matrixes for a multi-class system. The labels were generated according to the time prior to a seizure. The five labels were 0: 1-5 min before a seizure; 1: 5-65 min before a seizure; 2: 65 min to 8 hr before a seizure; 3: 8-24 hr before a seizure; 4: more than 24 hr before a seizure.

| **Patient 1** | | **Predicted** | | | | |
| --- | --- | --- | --- | --- | --- | --- |
|  |  | **0** | **1** | **2** | **3** | **4** |
| **Actual** | **0 (0.0005)** | 0.132 | 0.396 | 0.132 | 0.075 | 0.264 |
|  | **1 (0.0044)** | 0.030 | 0.392 | 0.225 | 0.061 | 0.292 |
|  | **2 (0.0304)** | 0.000 | 0.071 | 0.296 | 0.182 | 0.451 |
|  | **3 (0.0583)** | 0.006 | 0.108 | 0.048 | 0.327 | 0.512 |
|  | **4 (0.9064)** | 0.003 | 0.085 | 0.085 | 0.168 | 0.659 |
| **Time selected in predictions** | | 0.003 | 0.088 | 0.090 | 0.177 | 0.642 |
| **Proportion of total labels correct** | | 0.627 | | | | |

Table S13: The confusion matrix for Patient 1 when using a five label system. Values in the matrix are normalized along the actual rows. Values in the brackets in the actual labels indicate the proportion of actual labels in the test data.

| **Patient 2** | | **Predicted** | | | | |
| --- | --- | --- | --- | --- | --- | --- |
|  |  | **0** | **1** | **2** | **3** | **4** |
| **Actual** | **0 (0.0004)** | 0.290 | 0.355 | 0.000 | 0.065 | 0.290 |
|  | **1 (0.0029)** | 0.083 | 0.253 | 0.004 | 0.046 | 0.614 |
|  | **2 (0.0222)** | 0.004 | 0.102 | 0.008 | 0.026 | 0.860 |
|  | **3 (0.0425)** | 0.001 | 0.023 | 0.077 | 0.072 | 0.826 |
|  | **4 (0.9320)** | 0.001 | 0.022 | 0.037 | 0.100 | 0.840 |
| **Time selected in predictions** | | 0.001 | 0.025 | 0.038 | 0.097 | 0.839 |
| **Proportion of total labels correct** | | 0.787 | | | | |

Table S14: The confusion matrix for Patient 2 when using a five label system. Values in the matrix are normalized along the actual rows. Values in the brackets in the actual labels indicate the proportion of actual labels in the test data.

| **Patient 3** | | **Predicted** | | | | |
| --- | --- | --- | --- | --- | --- | --- |
|  |  | **0** | **1** | **2** | **3** | **4** |
| **Actual** | **0 (0.0115)** | 0.275 | 0.052 | 0.074 | 0.183 | 0.415 |
|  | **1 (0.0878)** | 0.227 | 0.055 | 0.067 | 0.248 | 0.404 |
|  | **2 (0.2914)** | 0.095 | 0.040 | 0.176 | 0.171 | 0.518 |
|  | **3 (0.3732)** | 0.054 | 0.040 | 0.200 | 0.294 | 0.412 |
|  | **4 (0.2360)** | 0.062 | 0.012 | 0.220 | 0.257 | 0.450 |
| **Time selected in predictions** | | 0.086 | 0.035 | 0.185 | 0.244 | 0.451 |
| **Proportion of total labels correct** | | 0.275 | | | | |

Table S15: The confusion matrix for Patient 3 when using a five label system. Values in the matrix are normalized along the actual rows. Values in the brackets in the actual labels indicate the proportion of actual labels in the test data.

| **Patient 6** | | **Predicted** | | | | |
| --- | --- | --- | --- | --- | --- | --- |
|  |  | **0** | **1** | **2** | **3** | **4** |
| **Actual** | **0 (0.0007)** | 0.033 | 0.083 | 0.300 | 0.283 | 0.300 |
|  | **1 (0.0070)** | 0.000 | 0.094 | 0.295 | 0.354 | 0.258 |
|  | **2 (0.0441)** | 0.014 | 0.105 | 0.364 | 0.265 | 0.253 |
|  | **3 (0.0884)** | 0.017 | 0.098 | 0.235 | 0.322 | 0.327 |
|  | **4 (0.8598)** | 0.009 | 0.052 | 0.122 | 0.197 | 0.620 |
| **Time selected in predictions** | | 0.010 | 0.059 | 0.144 | 0.212 | 0.575 |
| **Proportion of total labels correct** | | 0.58 | | | | |

Table S16: The confusion matrix for Patient 6 when using a five label system. Values in the matrix are normalized along the actual rows. Values in the brackets in the actual labels indicate the proportion of actual labels in the test data.

| **Patient 8** | | **Predicted** | | | | |
| --- | --- | --- | --- | --- | --- | --- |
|  |  | **0** | **1** | **2** | **3** | **4** |
| **Actual** | **0 (0.0039)** | 0.504 | 0.119 | 0.061 | 0.193 | 0.123 |
|  | **1 (0.0349)** | 0.389 | 0.191 | 0.161 | 0.155 | 0.105 |
|  | **2 (0.2074)** | 0.114 | 0.107 | 0.503 | 0.153 | 0.122 |
|  | **3 (0.2457)** | 0.090 | 0.085 | 0.270 | 0.478 | 0.077 |
|  | **4 (0.5081)** | 0.098 | 0.083 | 0.301 | 0.374 | 0.143 |
| **Time selected in predictions** | | 0.111 | 0.093 | 0.330 | 0.345 | 0.121 |
| **Proportion of total labels correct** | | 0.303 | | | | |

Table S17: The confusion matrix for Patient 8 when using a five label system. Values in the matrix are normalized along the actual rows. Values in the brackets in the actual labels indicate the proportion of actual labels in the test data.

| **Patient 9** | | **Predicted** | | | | |
| --- | --- | --- | --- | --- | --- | --- |
|  |  | **0** | **1** | **2** | **3** | **4** |
| **Actual** | **0 (0.0055)** | 0.434 | 0.368 | 0.179 | 0.019 | 0.000 |
|  | **1 (0.0560)** | 0.168 | 0.500 | 0.331 | 0.001 | 0.000 |
|  | **2 (0.2340)** | 0.044 | 0.135 | 0.816 | 0.005 | 0.000 |
|  | **3 (0.2543)** | 0.069 | 0.156 | 0.142 | 0.631 | 0.001 |
|  | **4 (0.4502)** | 0.066 | 0.151 | 0.435 | 0.344 | 0.004 |
| **Time selected in predictions** | | 0.069 | 0.169 | 0.443 | 0.317 | 0.002 |
| **Proportion of total labels correct** | | 0.384 | | | | |

Table S18: The confusion matrix for Patient 9 when using a five label system. Values in the matrix are normalized along the actual rows. Values in the brackets in the actual labels indicate the proportion of actual labels in the test data.

| **Patient 10** | | **Predicted** | | | | |
| --- | --- | --- | --- | --- | --- | --- |
|  |  | **0** | **1** | **2** | **3** | **4** |
| **Actual** | **0 (0.0035)** | 0.140 | 0.201 | 0.156 | 0.419 | 0.084 |
|  | **1 (0.0432)** | 0.034 | 0.477 | 0.128 | 0.319 | 0.040 |
|  | **2 (0.2573)** | 0.012 | 0.144 | 0.286 | 0.496 | 0.061 |
|  | **3 (0.3262)** | 0.011 | 0.062 | 0.076 | 0.769 | 0.082 |
|  | **4 (0.3698)** | 0.006 | 0.078 | 0.107 | 0.498 | 0.312 |
| **Time selected in predictions** | | 0.011 | 0.108 | 0.144 | 0.578 | 0.160 |
| **Proportion of total labels correct** | | 0.461 | | | | |

Table S19: The confusion matrix for Patient 10 when using a five label system. Values in the matrix are normalized along the actual rows. Values in the brackets in the actual labels indicate the proportion of actual labels in the test data.

| **Patient 11** | | **Predicted** | | | | |
| --- | --- | --- | --- | --- | --- | --- |
|  |  | **0** | **1** | **2** | **3** | **4** |
| **Actual** | **0 (0.0036)** | 0.353 | 0.434 | 0.030 | 0.136 | 0.047 |
|  | **1 (0.0305)** | 0.126 | 0.482 | 0.138 | 0.151 | 0.103 |
|  | **2 (0.1515)** | 0.045 | 0.277 | 0.451 | 0.118 | 0.109 |
|  | **3 (0.2918)** | 0.023 | 0.075 | 0.183 | 0.576 | 0.143 |
|  | **4 (0.5227)** | 0.028 | 0.174 | 0.260 | 0.350 | 0.188 |
| **Time selected in predictions** | | 0.033 | 0.171 | 0.262 | 0.374 | 0.160 |
| **Proportion of total labels correct** | | 0.350 | | | | |

Table S20: The confusion matrix for Patient 11 when using a five label system. Values in the matrix are normalized along the actual rows. Values in the brackets in the actual labels indicate the proportion of actual labels in the test data.

| **Patient 13** | | **Predicted** | | | | |
| --- | --- | --- | --- | --- | --- | --- |
|  |  | **0** | **1** | **2** | **3** | **4** |
| **Actual** | **0 (0.0023)** | 0.631 | 0.110 | 0.020 | 0.143 | 0.096 |
|  | **1 (0.0203)** | 0.156 | 0.414 | 0.082 | 0.188 | 0.160 |
|  | **2 (0.1265)** | 0.076 | 0.301 | 0.412 | 0.108 | 0.103 |
|  | **3 (0.2464)** | 0.081 | 0.060 | 0.201 | 0.363 | 0.295 |
|  | **4 (0.6045)** | 0.093 | 0.106 | 0.238 | 0.279 | 0.284 |
| **Time selected in predictions** | | 0.091 | 0.125 | 0.247 | 0.276 | 0.261 |
| **Proportion of total labels correct** | | 0.323 | | | | |

Table S21: The confusion matrix for Patient 13 when using a five label system. Values in the matrix are normalized along the actual rows. Values in the brackets in the actual labels indicate the proportion of actual labels in the test data.

| **Patient 15** | | **Predicted** | | | | |
| --- | --- | --- | --- | --- | --- | --- |
|  |  | **0** | **1** | **2** | **3** | **4** |
| **Actual** | **0 (0.0009)** | 0.038 | 0.000 | 0.013 | 0.064 | 0.885 |
|  | **1 (0.0088)** | 0.001 | 0.033 | 0.013 | 0.055 | 0.898 |
|  | **2 (0.0553)** | 0.001 | 0.022 | 0.022 | 0.008 | 0.948 |
|  | **3 (0.1236)** | 0.000 | 0.017 | 0.001 | 0.000 | 0.981 |
|  | **4 (0.8114)** | 0.000 | 0.010 | 0.029 | 0.036 | 0.926 |
| **Time selected in predictions** | | 0.0004 | 0.012 | 0.025 | 0.030 | 0.933 |
| **Proportion of total labels correct** | | 0.753 | | | | |

Table S22: The confusion matrix for Patient 15 when using a five label system. Values in the matrix are normalized along the actual rows. Values in the brackets in the actual labels indicate the proportion of actual labels in the test data.
